# Are Virtual Particles Less Real?

**DOI:** 10.3390/e21020141

**Published:** 2019-02-02

**Authors:** Gregg Jaeger

**Affiliations:** Quantum Communication and Measurement Laboratory, Department of Electrical and Computer Engineering and Division of Natural Science and Mathematics, Boston University, Boston, MA 02215, USA; jaeger@bu.edu

**Keywords:** quantum field, elementary particle, quantum, scattering, perturbation theory, ontology, virtual particle

## Abstract

The question of whether virtual quantum particles exist is considered here in light of previous critical analysis and under the assumption that there are particles in the world as described by quantum field theory. The relationship of the classification of particles to quantum-field-theoretic calculations and the diagrammatic aids that are often used in them is clarified. It is pointed out that the distinction between virtual particles and others and, therefore, judgments regarding their reality have been made on basis of these methods rather than on their physical characteristics. As such, it has obscured the question of their existence. It is here argued that the most influential arguments against the existence of virtual particles but not other particles fail because they either are arguments against the existence of particles in general rather than virtual particles *per se*, or are dependent on the imposition of classical intuitions on quantum systems, or are simply beside the point. Several reasons are then provided for considering virtual particles real, such as their descriptive, explanatory, and predictive value, and a clearer characterization of virtuality—one in terms of intermediate states—that also applies beyond perturbation theory is provided. It is also pointed out that in the role of force mediators, they serve to preclude action-at-a-distance between interacting particles. For these reasons, it is concluded that virtual particles are as real as other quantum particles.

## 1. Introduction

The notion of the virtual particle arises in the context of relativistic quantum field theory where elementary particles are considered quanta associated with fields characterized via appropriate Hamiltonian or Lagrangian functions. Even though virtual particles have been and continue to be widely used in descriptions, explanations, and predictions of fundamental physical phenomena at subatomic scales, the reality of the virtual particles *as distinguished from free particles* has been the subject of an ongoing debate leaning toward their dismissal—cf. [1] Section 3 and [2,3] for summaries.

The standard distinction between virtual and other particles is made within a particular approach—that of Feynman diagrams—to the calculation of perturbation-theoretical quantities that are only indirectly related to the observed events to which they may, but need not be applied. The particles that are in practice (relatively) directly prepared, detected and thereby observed are, in standard analyses, reducible to fields taken to be free in the initial and the final, large-time limits; this is not so for the virtual particles, which are considered to mediate interactions between other particles taking place *between* these time limits when *none* of the particles involved can be accurately considered free. The fact that the particles labeled virtual are those not later directly observed in the already very limited sense that other particles are said to be is sometimes in itself taken as a ground for skepticism toward their existence. However, being directly observed free is not a necessary condition for existence. For example, in particle physics, the existence of quarks is rarely questioned, at least not vigorously, even though are not so observed. (See [4] for a discussion of the sense in which quarks can be observed). Indeed, quarks are widely regarded as fundamental building blocks of matter of which hadrons are (mainly) constituted, are believed to be confined within those composite particles, and have never appeared (at least convincingly) in isolation. (Cf., e.g., [5], Chapter 8. Note also that as a result, quarks and the gluons that bind them do not correspond to “poles”, unlike other non-virtual particles).

Nonetheless, it has been argued that virtual particles do not exist even when the other sort, the so-called ordinary particles, do exist. The powerful arguments against the reduction of fundamental physics to that of particles in the traditional sense of that notion, rather than fields, are not addressed here; see [6,7] for discussion of their status as a class. (As discussed below, a good criterion for holding that individual instances of particles, as understood here, are non-present is that the corresponding field mode has zero probability of having been appropriately excited. Note also that because the “quantum field” is operator-valued, calling a field “excited” is short hand to indicate that the state in question is such that energy is added. In quantum field theory (QFT) and particle physics, a particle is standardly called “quantum field excitation”, a term used as a *façon de parler* for an energy quantum with specific characteristics and “excited field” for a state corresponding to having quanta added). Here, after discussions of the distinction of virtual from other particles and of the theoretical and mathematical elements involved in explanations in particle physics, I show that the most influential such arguments against virtual particles fail because: (i) they impose inappropriate requirements, or (ii) they assume a distinction between virtual and other particles that depends on the choice of analytical perspective taken toward their behavior, which in turn varies according to the physical circumstances of the particles—for example, distance of propagation and (non-)interaction with other particles within the time interval involved—or (iii) they are beside the point. Whether a given sort of particle either exists or does not exist must be decided independently of the analytical approach to studying the behavior of the associated quantum fields. It is argued here that virtual particles exist, as do other more directly observed particles, because their presence helps explain observed phenomena. (To assert that something exists, it is taken here as sufficient that it gives rise to observable effects, directly or indirectly. See also [7] (Chapter 4) and [8] (Section 1.2.2) regarding realism). It is noted that their existence also allows, in the theoretical context, for the preservation of local causality, given that distant particles interact. (Cf. [9] (Section 1.3) and the comments of Steven Weinberg near the end of the following section).

## 2. The Real/Virtual Distinction and its Limits

The distinction between virtual quanta and other quanta is standardly made in a very specific way: the virtual particles are those that appear as “internal lines” of the Feynman diagrams; and any particle not eventually appearing as a quantum of a state of a free field is represented by an internal line. These diagrams are primarily computational tools productively used to represent the various (infinite in number) terms appearing in perturbation-theoretical calculations of outcomes of the scattering, for example, in the experiments that are the empirical mainstay of modern particle physics; these calculations are made more convenient by (optionally) using this form of diagram as an accounting aid in perturbation-theoretical methods for the calculation of relevant quantities.

However, for any sort of particle that can appear free, there are circumstances in which a particle of exactly same sort (as defined by inherent properties, discussed below) that is certainly present because of its having a measurable influence on systems (that only happen to be other than (parts of) particle detectors) will be classified as virtual: An instance of what would have been considered an ordinary particle could by the above standard be classified as virtual only because it happened to “run into” another object and be absorbed by it before detection. (N.b. One must recognize the need for treading carefully here, as a counterfactual aspect is involved). For example, a photon may run into an electron instead of being detected at some later time; a photon can be absorbed by an electromagnetically charged particle at *any time or distance whatsoever* (due to its masslessness)—even billions of light-years from its ostensible location of emission, cf., e.g., the Wheeler delayed-choice experiment [10]—instead of being detected in an interaction with a measuring instrument, say, immediately previously or immediately later, and so be represented by an internal line in a Feynman diagram (rather than an external line) that may correspond to the most numerically significant contribution to the calculated values appearing in an expeditious calculation used in an explanation of the event.

In addition, given sufficient energy, it is possible to produce *any* such particle as a virtual one via the process of pair creation, that is, the production of a particle together with its antiparticle, when more than twice the rest mass associated with its sort sort of field is available. In the exotic circumstances of pair creation at the event horizon of a black hole, it is even possible for one of the two “sister" particles to escape the black hole as a free particle but the other to disappear below the horizon, leaving the former “behind”. (This is the mechanism seen as giving rise over the *very* long run to black-hole “evaporation”, the running of its mass to below the requirement from being such an object). A more ordinary example of pair creation, which regularly occurs in the context of experimental high-energy physics, is in an electromagnetic shower, something used for particle measurement in electromagnetic calorimetry, e.g., the measurement of the energy of a high-energy electron through the use of an appropriate medium (for example, one involving atoms of high atomic number, a “high-*Z*” medium): In such circumstances, the electron very likely emits a (virtual or non-virtual) bremsstrahlung photon, itself subsequently capable of producing (virtual) electron-position pairs which produce further photons (virtual or non-virtual), with the subsequent excited-electron states continuing to emit (virtual or non-virtual) bremsstrahlung photons, and so on in a cascade until the emitted photon average energy falls below a critical energy value—after which the electrons and photons continue to lose energy via other, ionization processes; cf. e.g., [11], Section 1.2. In fact, the process producing bremsstrahlung can occur for any sort of charged particle, with a rate of production within the shower that is inversely proportional to the square of the mass of the particle; all these possibilities have a priori and a posteriori probabilities that relate to what is observed.

Moreover, similar particle showers can be produced via *nuclear* rather than electromagnetic interactions by high-energy hadrons entering an appropriate medium; in a typical large particle detector, one finds a tracking system (a silicon-strip detector of “pixels”, wherein a charged particle traversing an appropriately doped silicon wafer gives rise to up to $10^{4}$ electron–hole pairs by ionization, providing precise 2D spatial positions) in a magnetic field (assisting in charge detection via the influence of Lorentz force), followed by an electromagnetic calorimeter (ECAL), a hadron calorimeter (HCAL), and muon detectors ([11], Section 1.3).

It is also sometimes, though less often, taken instead as the distinguishing characteristic of a virtual particle that it be “off mass-shell”, that is, that it have a mass not corresponding to the value standardly attributed to the corresponding quantum and, so, be a virtual version of that quantum. This may or may not coincide with its being symbolized by an internal line in perturbation-theoretical diagrams and is also a consequence of electing a particular mathematical method of analysis, that of Feynman himself—this is discussed near the end of Section 5, below. For now, note that in Feynman’s approach, if within these diagrams internal lines are related to particles being taken to have values varying from their standardly attributed masses, the satisfaction of an energy conservation rule is ensured even ‘where’ the lines that represent particles intersect; the deviation from the standard mass is seen to approach zero as the spatial separation is increased between the interacting particles. Alternatively, the mass can be taken to be fixed at its standard value, the corresponding energy-momentum relation—$E^{2}=p^{2}+m^{2},$ where *p* is momentum, *m* is mass, and c=1—may be considered not to be satisfied at the diagrammatic vertices, as discussed in greater detail in Section 5, below, instead of enforcing this relation. (It is because this equation is that of a sphere that particles not satisfying it are considered *off* shell, that is, off that sphere). It is, therefore, sometimes instead (incorrectly) concluded that virtual particles violate the conservation of energy. However, this conclusion is reached based on the use of a classical (even non-Lorentz-covariant) understanding of physical processes partially (and in a very abstract sense) ‘portrayed’ via different specific sorts of perturbation-theoretic diagrams and/or the use of an overly strict enforcement of conservation of energy at the expense of Lorentz covariance—see [9], pp. 151–155 and the end of Section 5.

The consideration of quanta as off mass-shell, like the perturbation-theoretical calculations in which it arises, is therefore a matter of the choice of method of analysis of interaction and, more importantly, neither of the above alternatives requires the rejection of virtual quanta as quanta, that is, to consider them not to exist in the sense I am arguing here that they do.

The most influential argument against the reality of virtual particles, which has come to be known as the “superposition argument”, is that virtual particles do not exist because neither the number nor kinds of virtual particles are determinate during scattering processes as modeled by quantum field theory—cf. [1,12] for statements of its slightly differing formulations. As noted by its advocates, this situation is analogous to that in simple non-relativistic quantum mechanics when, as is often the case, the system state is a superposition of eigenstates of a given observable of interest, for the simple reason that particle number is a quantum observable in (second-quantized) quantum field theory; yet, there is little question that the systems of quantum mechanics do exist and possess properties, however non-classically or unsharply. Indeed, the argument applies equally well to non-virtual particles (as aspects of the states of the quantum field) except at the single moment of their detection in the asymptotic time limits when the associated values may be momentarily sharpened by measurement. This argument is also considered further in Section 5 below.

With this brief review of the grounds offered for skepticism toward the existence of virtual particles and brief retorts to them in mind, let us also note that there are good independent grounds for asserting the existence of virtual particles. Virtual particles appear in successful physical explanations—indeed, in some of the most precisely confirmed predictions of in the history of science; they are also invoked in descriptions and explanations of physical situations of great significance for the development of recent physics, including those just mentioned, by some of the most accomplished physicists of recent history, who are therefore among those in the best position to evaluate their descriptive and explanatory value and who (in some cases) explicitly endorse their reality on that ground.

A clear example of this can be found in the 1988 Dirac Memorial Lectures of Abdus Salam, who systematically refers to the virtual particles which he, like others, calls “messengers” to reflect their role in mediating fundamental forces: “In quantum theory, all forces—whether gauge or non-gauge variety—are produced by an exchange of particles, which I shall call ‘messengers’ ” ([13], p. 39) because, by serving as local agents mediating between scattered particles, these particles serve to uphold the prohibition of action-at-distance, something that is a problematic feature of the use of the classical Coulomb’s law. (Recall that the very act of mediating “between” two other particles is what standardly classifies a particle as virtual by virtue of how such “transmission” is then represented in diagrams serving as aids in the calculation of probabilities of the occurrence of such (sub-)processes).

The following commentary from Steven Weinberg’s 1977 article “A Search for Unity: Notes for a History of Quantum Field Theory” indicates the significance of the introduction of virtual particles by quantum field theory and addresses this as well as the issue of energy conservation: “It is important to understand that quantum field theory gave rise to a new view not only of particles but also of the forces among them. We can think of two charged particles interacting at a distance not by creating classical electromagnetic fields which act on one another, but by exchanging photons, which continually pass from one particle to the other. Similarly, other kinds of force can be produced by exchanging other kinds of particle. These exchanged particles are called virtual particles, and are not directly observable while they are being exchanged, because their creation as real (i.e., free) particles (e.g., a free electron turning into a photon and an electron) would violate the law of conservation of energy. However, the quantum-mechanical uncertainty principle dictates that the energy of a system that survives for only a short time must be correspondingly highly uncertain, so these virtual particles can be created in intermediate states of physical processes, but must be reabsorbed again very quickly” [14]. A crucial thing to note here is Weinberg’s reminder of the caveat against strict, classical (i.e., zero uncertainty) enforcement of the *equation* (Equation (4), below) appearing in the conservation law—the grounds cited for “violation of the law of conservation of energy”—that in quantum theory is related to quantum energy indeterminacy, about which cf. [15].

In addition to their role as force-mediating particles, the presence of virtual particles in relation to the important processes of pair-production is also recognized as explanatory. For example, Julian Schwinger commented on his early work with J. Robert Oppenheimer that it “used quantum electrodynamics to describe the electron-positron emission from an excited oxygen nucleus, which emphasized for me the physical reality of such virtual photon processes” ([16], p. 330). It has also been discussed more recently by Gordon Kane who commented that “Virtual particles are indeed real particles.…one particle can become a pair of heavier particles (the so-called virtual particles), which quickly rejoin into the original particle as if they had never been there. If that were all that occurred we would still be confident that it was a real effect …However, while the virtual particles are briefly part of our world they can interact with other particles, and that leads to several tests of the quantum-mechanical predictions about virtual particles”, such as the “Lamb shift..., for which a Nobel Prize was eventually awarded” [17]. (N.b. Although much of the discussion below centers around scattering processes, note that neither the Lamb shift nor the anomalous magnetic moment of the electron (“*g*-2”) pertain only in the sort of scattering performed in collision experiments).

## 3. QFT, Particle Physics, and Feynman Diagrams

Because arguments both for and against the existence of virtual particles crucially involve the methods of application of the mathematical formalism of quantum field theory and their connection to measurement and observation, their elements are now described. The arguments themselves, mentioned in the introduction, are handled more precisely than above in following sections.

Short-lived, virtual particles are considered in predictions and explanations of the primary occurrences of interest to modern particles physics because they contribute to observed effects. These predictions and explanations are made based on calculations typically, though not necessarily, involving perturbation-theoretical methods. They involve finding the probabilities for the various possible overall processes between relatively directly observed initial and possible final states. The presence of an elementary particle is typically inferred after the fact based on constellations of measurements of field properties, such as charge and momentum, in specific space-time regions. These properties can be ascertained from, for example, relatively large-scale tracks (in trackers of the sort described in Section 2) or directly by appropriate detectors (such as the calorimeter banks also described there) targeting the properties of interest. In these processes, which include production, absorption, or decay, particles may scatter from each other and/or come and go out of existence. Importantly for several of the arguments considered in later sections, the calculations involved can often be aided by diagrams, corresponding the possible ways for relevant scattering sub-processes, used for accounting.

In experiments, elementary particles are typically injected into a cyclotron (e.g., E. O. Lawrence’s) or a particle collider (e.g., the Large Hadron Collider), considered to be scattered via fundamental forces from one another and possibly other intervening particles, and then measured by registration in purpose-built apparatus. The following textbook summaries describe how this is standardly conceived and approached. “At a particle accelerator, the colliding beams produce individual interactions referred to as events. The large particle physics detector systems use a wide range of technologies to detect and measure the properties of the particles produced in these high-energy collisions with the aim of reconstructing the primary particles produced in the interaction. In essence, one tries to go from the signals in the different detector systems back to the Feynman diagram responsible for the interaction” ([11], p. 22). “[S]cattering experiments have been a fruitful and efficient way to determine the particles that exist in nature and how they interact. In a typical collider experiment, two particles, generally in approximate momentum eigenstates at [some initial time, approximated well by] t=−∞, are collided with each other and we measure the probability of finding particular outgoing momentum eigenstates at t=+∞. All the interesting interacting physics is encoded in how often given initial states produce given final states, that is, in the S-matrix” [18], Chapter 6. Notably, “the particles produced in these high-energy collisions” which are the very focus of those experiments constructed “to determine the particles that exist” are, in fact, mostly the *virtual* versions of particles because to be discovered they must, in the terrestrial environment, be created intentionally and, due to their short-livedness, will decay by the time the final (to a good approximation, free) scattered state is measured.

Cross-sections and decay rates are the empirical quantities of greatest interest in such experiments. In physical analyses of scattering, the intuitive, common-sense notion of area is replaced by the physicist’s cross-section, a quantity having units of area but considered to serve as a measure of interaction strength rather than a literal area of collision. Again, because quantum mechanics provides only probabilities of occurrences, the cross-section σ is usually considered in the (preferably readily integrable) form

(1) $$d\sigma =\frac{1}{T}\frac{1}{\Phi }dP\;,$$

where *P* is the probability for the transition between the two states (the initial prepared state and that found in the final measurement) and Φ is the normalized flux; *P* and σ are here considered in their differential form (in angle, energy, etc., as convenient). Scattering data is typically provided in terms of numbers of events measured, which (again, in differential form) is dN=Ldσ, where *L* is a proportionality factor known as the luminosity; in QFT, *P* is directly related to the appropriate S-matrix element.

Any scattering or decay process of particles is describable as a transition between initial and final energy–momentum eigenstates and takes place with the involvement of short-lived quanta, that is, virtual particles in the intermediary states. For example, the (electromagnetic) scattering of two initially free electrons can be viewed as involving the transmission of at least one virtual photon, $\gamma^{*}$. In the case of decay processes, such as the $\beta^{-}$-decay of a neutron (n→ e${}^{-}$+p+${\bar{\nu }}_{e}$), one typically considers a single, initial particle to give rise to *n* particles within a finite time-duration *T*, so that from the mathematical point of view the process can be treated in the same way as the scattering of a single particle with the vacuum state (of no particles) to an *n* particle final state (cf. e.g., [18], Section 5.1.1), in this case via the electroweak force involving an intermediate (virtual) W${}^{-}$ particle. Thus, all observed phenomena of particle physics can, within the constraints of such approximations, be related to the quantum fields appearing in theory through experiments involving standard preparation and detection procedures and will involve (virtual quanta of) force-mediating fields.

The above quantities relating to detection rates, calculable given the specification of preparation procedures, arise in QFT in terms of probabilities of the form $|\langle f;t_{f}|i;t_{i}\rangle {|}^{2}$, where *i* and *f* indicate initial and final measured states and $t_{j}$ times, here considered in the relatively intuitive, so-called Schrödinger ‘picture’ in which it is the state alone that is understood to evolve in time. (Pictures are discussed further shortly below). The theory currently regarded as basic to particle physics is Relativistic Quantum Field Theory (RQFT), with Quantum Electrodynamics (QED) continuing to serve as its archetype; cf. [19]. In RQFT, each sort of elementary particle can be defined, for example, group-theoretically in the manner of Eugene Wigner as the physical model of the irreducible representation of the symmetry group associated with a corresponding field—in which particles are the quanta, that is, countable energy units which can be systematically accounted for—via solutions of the law of motions governing it [20]. In standard RQFT, the prepared and measured particles are viewed as *field quanta*: units corresponding to contributions to the eigenvalues of energy associated with a specific field, itself distinguished by unique combined values of mass, charge, and spin, say, as above. Particles are most easily observed as single-excitation-sized energy contributions in regions sufficiently far from regions of interaction with those of other fields to be considered effectively free, when the involvement of forces can be taken to be effectively limited in space and time, although the electromagnetic force of QED has infinite range, requiring this to be only approximately so.

Each particle or set of particles is thus a local aspect of the quantum state ([6], Section 6.4.2) and is subject to statistical constraints. The always probabilistic-statistical calculations of the observable quantities are such that the temporal details of the interactions are left unspecified and involve all of space and time, although the fields involved are considered to interact locally and in time when they do; all possible alternatives consistent with the given set of initial and final states are incorporated in any concrete situation. However, because of quantum fluctuations, it is generally difficult to give precise field strengths to specific *points* or to give sharp intensities in specific regions of space-time in RQFT. Hence, as Victor Weisskopf put it, “the days of fixed particle numbers are over. Particles must be considered as the quanta of a field, just as photons are the quanta of the electromagnetic field; such quanta are created or destroyed. The theory of the interaction of charged particles with the radiation field has become a field theory, a theory in which two (or more) quantized fields interact: the matter field(s) and the radiation field” ([16], pp. 66–67).

Nonetheless, initial strength and intensity of a field can be attributed based on local preparations with the field conceived of as a collection of modes, and these have precise average values. Incoming and outgoing eigenstates and respective quanta are commonly and relatively uncontroversially considered real by practitioners, even when the initial state is taken to be the lowest energy, so-called “vacuum state”; intermediate states are also often viewed this way, though more controversially so, particularly when the mathematical correspondents of very short-lived particles appear, which they always do when fields are considered to interact without action-at-a-distance.

As mentioned above, for the purposes of understanding interactions, quantum systems are viewed as prepared in specific energy-momentum eigenstates |i〉 at some initial time $t=t_{i}$ and involved in scattering processes, after which, at the final time considered, particles are found away from the interaction region when the state |f〉 is measured. However, quantum-field-theoretical calculations are often performed using the so-called Heisenberg “picture” (convention) wherein the operators rather than states are taken to depend on time, as is the case in the more intuitive Schrödinger picture, for convenience and with the same final results. The time-evolving operator for this picture is the S-matrix *S*, definable as

(2) $${\langle f|S|i\rangle }_{H}={\langle f;t_{f}|i;t_{i}\rangle }_{S}\;,$$

where here the state subscripts indicate the convention used—H for the Heisenberg and S for the Schrödinger—under the assumption that all possible changes occur within a finite time and $t_{f}$ corresponds to the large-time limit t→+∞, so that such field equation solutions (the asymptotic states of the field) can be considered free before and after the interaction period; cf. ibid.. The final state is that detected beyond the (finite or approximately finite) interaction region.

Now, it is significant for some of the arguments considered here that not only can these initial and final states |i〉 and 〈f| be expressed in terms of having had the raising and lower operators ${{\hat{a}}_{k}}^{†},{\hat{a}}_{k}$, respectively, applied mathematically to the lowest-energy (“vacuum”) states involved, but also intermediate states (possible states between these time limits), so that states involving virtual particles (i.e., field excitations) can be written with the same operators applied to the initial and (other, meanwhile arising) intermediate states. The intermediate quantum states considered in scattering can also be represented mathematically by the application of factors known as field “propagators” appearing in integrals summed to provide values for the scattering-matrix elements: The S-matrix elements can be represented by a mathematical expansion into sums of terms—the scattering amplitudes—in the perturbation-theoretical approach, where one considers mathematical expansions in powers of factors that each can be associated with the amount of interaction taking place between the initial and final times. It is also significant that the substantially greatest contributions to these sums are typically those of the lower orders in the expansion—because simpler interactions are generally more likely—and, so, often receive exceptional attention. Note also that the initial state (with incoming quanta) and final state (with outgoing quanta) with interacting particles (“coupled” fields) involved in the description of scattering are of the same sort considered in situations where no interaction takes place.

The elements of the S-matrix are transition probabilities (the squares of the quantum state amplitudes) connecting the initial and final states, each proportional to the corresponding differential cross-section, cf. Equation (1). These can be conveniently calculated, for example, via the Lehmann– Symanzik–Zimmermann (LSZ) reduction formula (which, among other things, naturally incorporates time-ordering of interactions with the assistance of the time-ordering T{·}, which directly orders in time, from right to left, the field operators involved in possible interactions):

(3) $$\langle f|S|i\rangle \propto \langle \Omega |T\left\{\phi \left(x_{1}\right)\phi \left(x_{2}\right)\ldots \phi \left(x_{n}\right)\right\}|\Omega \rangle \;,$$

where the $\phi (x_{i})$ are the operators corresponding to the interacting fields and |Ω〉 is the vacuum (ground state) of the theory including interacting fields; cf. [18], Section 6.1. The calculation of the values provided by the associated sums can be eased using diagrams, such as Feynman diagrams, which are composed of lines that can be viewed as symbolizing particle propagation, with intersections (“vertices”) symbolizing possible mediated interactions between the fields involved: incoming and outgoing states are represented by incoming and outgoing lines, ordered in time, and to each internal line there corresponds a Green’s function as its propagator. (Propagators are called so because their classical correspondents characterize the propagation of a field through space when a corresponding current—say of a charge—acts as a source). When integrated, the products of the Green’s functions appearing in terms of the perturbative expansion of S-matrix elements provide the transition probability contributions corresponding to various Feynman diagrams.

Every Feynman diagram is constructed according to a specific set of rules (one for position-space calculations, one for momentum-space calculations), and each represents a possible physical process with the same incoming and outgoing states; all possible processes between these states are taken into account by adding these various contributions together. There will be internal lines in any non-trivial process, that is, lines corresponding to neither incoming nor outgoing quanta but to the short-lived (virtual) particles. The Feynman rules for performing momentum-space calculations, which are typically preferred over the position-space version as a matter of convenience, are six (cf. [18], p. 95):

(1) Each internal line symbolizes a propagator of the form
  $${\left(p^{2}-m^{2}+i\epsilon \right)}^{-1},$$
  where *p* is the four-momentum, *m* the particle mass, and c=1;
(2) Each vertex symbolizes an imaginary factor with the magnitude of the appropriate coupling constant;
(3) Lines connected to external points require no propagator (as they are cancelled out, as discussed further above);
(4) Momentum conservation is imposed at each vertex;
(5) Undetermined four-momenta are integrated over;
(6) All possible diagrams (each an integral) are summed.

Again, it is important to remember that these diagrams are only abstract symbolic representations and computational aids; although highly convenient, they are not necessary for the performance of perturbation-theoretical calculations; and, despite appearances, the lines appearing in these diagrams are not specific space-time trajectories—in fact, *all of space* is considered (integrated over) in the calculations to which they correspond, not just a single possible trajectory [21,22]. (Note also that Feynman-like diagrams are also used as tools for performing calculations other than perturbative ones; cf. [23], Chapter 8 for a discussion of their various uses in practice).

This immediately brings into question the meaningfulness of any ontological distinction of virtual particles from other particles because it is (standardly) made based on how a given particle appears in these diagrams rather than directly to the quantum system state. Although a way of avoiding this particular form of the problem might seem to be offered by simply redefining the virtual particle as one corresponding to a propagator in the perturbation-theoretical calculation of field interactions independently of diagrammatics, even then the designation ‘virtual’ still depends on the use of that (extremely helpful but, ultimately, optional) calculation technique.

## 4. The Positions

With the practical and theoretical context of the standard distinction between virtual particles and other particles sketched above and the associated calculational and diagrammatic techniques in mind, let us now consider possible positions regarding existential status of virtual elementary particles: (I) there are no particles at all (including virtual ones); (II) there are particles but no *virtual* particles; (III) there are also virtual particles, but these have only an operational meaning; (IV${}^{\prime }$) virtual particles exist essentially in the same way as do their standard counterparts.

The first position is not engaged in this paper. However, because its assertion—for example by Michael Redhead (and others, cf. e.g., [7,24])—involves either the carrying over of implausible characteristics of classical particles to quantum particles or arguing it to be incompatible with quantum field theory, it is largely irrelevant to the issue at hand. (Recall that the current analysis is provided conditionally on the assumption of the existence of *some* particles, appropriately conceived, in particular, as quanta of energy associated with respective quantum fields). The second position has been repeatedly taken in the related literature and is relevant here; cf. the careful review by Tobias Fox in which this is made clear [1]. The arguments for this position are considered in detail in the following section, where it is argued that they fail to tell against the position defended here, that of (IV) offered below. The third view is that virtual quanta “are nothing but formal tools in the calculation of the interactions of quantum fields …”, which has been explored in detail by Brigitte Falkenburg who adds that “This does not mean, however, that the perturbation expansion of the S-matrix in terms of virtual particles is *completely* fictitious. The virtual processes described in terms of the emission and absorption of virtual particles contribute to a scattering amplitude or transition probability. Hence, *infinitely* many virtual particles *together* may be considered to cause a *real collective effect.* In this sense, they obviously *have* operational meaning” [6], Section 6.4. Related to this cautious position is the concern that that “unphysical aspects” can be wrongly attributed to scattering amplitudes if they are incorrectly composed or taken out of context, such as when partial scattering amplitudes are substituted for total quantum amplitudes in a manner not supported by an appropriate physical approximation. The arguments offered below for the reality of virtual particles are also sensitive to this concern.

The position defended here is a specific version of the fourth position (IV${}^{\prime }$): (IV) there are virtual particles in the same way that there are particles in general, namely, as aspects of the quantum state vis-à-vis associated quantum fields. Given that there is a major subfield of physics known as particle physics, the theoretical support for which is provided by quantum field theory, it is, a priori, a reasonable assumption that there do exist quantum particles in this sense, so the more interesting aspect of the position is that regarding virtual particles in particular. One strong support for IV is that the consideration of quanta as virtual is due to the choice of diagrammatics and mathematical analyses applied to the situations considered, as noted in Section 2, rather than the properties of the particles themselves understood as aspects of the field having the same fundamental characteristics as non-virtual particles and differing from them primarily by not being detected as free. Another point in favor of IV is that when it is physically legitimate to consider individual contributions to the scattering-matrix elements—as it is appropriate to do in situations where these have an overwhelmingly dominant significance to the observed behavior—powerful explanations of the associated phenomena similar to those preceding QFT can be given in terms of the same finite-lived particles (e.g., photons).

The debate surrounding the existence of virtual particles has taken place almost exclusively in the context where individual Feynman diagrams are improperly understood, namely, as detailed space-time diagrams with significant intuitive power. However, for example, analyses of effects involving collections of virtual particles giving rise to mass- and charge-value corrections—in which there are an infinite number of perturbation-theoretical contributions wherein more than one of them contributes significantly—are far less susceptible to such intuitive, quasi-physical misinterpretation but still involve intermediate quantum states including such quanta. Thus, simplistic treatments centered on individual diagrams have led the discussion astray; it should not be forgotten that particles in general, as aspects of quantum fields, are independent of diagrams and of the perturbation-theoretical context where they are almost exclusively used.

## 5. The Arguments Against Virtual Particles

Now, let us consider the most influential arguments against the existence of virtual particles: (i) a counterargument to a reconstructed “operator argument”, focused on an erroneous understanding of the meaning of the application of raising and lowering operators to state vectors, and (ii) the superposition argument, focused on ontological implications of the vectorial character of the quantum state. The most influential versions of these two arguments are those given by Robert Weingard and Paul Teller, which are considered here and followed by a presentation of reasons for asserting the existence of virtual particles. Here, Argument (i) is shown to be beside the point, and Argument (ii), although directed at virtual particles, is seen to pertain no more to virtual particles than to any other sort of particle, or indeed than to any quantum system. (Thus, Weingard’s discussion of the latter played a role in convincing Redhead to reject his own (extended) particle interpretation of QFT: “QFT is best understood in terms of quantized excitations of a field and that is all there is to it” [24], pp. 17–18, something not disputed here, but also something without direct implication for the position defended here, Position IV. N.b. The exclusively field interpretation has itself been subject to critique, cf. e.g., [25]).

Weingard offered Argument (i), that against the taking of the virtual particles to exist on the grounds that the raising and lowering operators appearing in the perturbation expansion of the elements of the S-matrix in calculations of amplitudes—for example, the amplitude for electromagnetic scattering of an electron by an electron—are the very same as those representing the creation of ordinary particle states from the vacuum state, that is, those of the input and output states just because “it seems natural…to interpret [these operators] as also creating and destroying ‘real’ photons in this case too… ”; correctly, he immediately dismisses such an inference.

To better understand what is involved, consider the rejected argument as formulated by Fox. “The reasoning goes as follows: as long as real particles enter our reality due to the application of creation operators, virtual particles ‘created’ in the same way should be treated equally” [1], p. 46. Such an argument is indeed readily dismissible: the *creation of particles* is not described by the simple application of raising *operators*; their “application” is a way of performing calculations of probability amplitudes and not directly interpretable as something physical; moreover, such a formal procedure need not even be carried out. For example, one could mathematically construct the representative of a single-photon input field state of definite momentum k/ℏ and polarization α by applying the operator $a_{k,\alpha }^{†}$ to the vacuum state |0〉 to obtain $a_{k,\alpha }^{†}|0\rangle$, thereby providing the eigenstate of the number operator $N_{k,\alpha }=a_{k,\alpha }^{†}a_{k,\alpha }$ corresponding to eigenvalue one, i.e.,

$$N_{k,\alpha }|1_{k,\alpha }\rangle =1|1_{k,\alpha }\rangle \;,$$

cf. [26], Section 2.2, instead of writing this state directly; but, this would represent no physical *preparation process* because manners of constructing mathematical representations of states and the processes by which they arise physically are neither identical nor isomorphic. (Another, more basic example to consider is that of the mixed quantum states, which can be mathematically decomposed in different, operationally inequivalent ways). Indeed, considering such a thing in itself as directly reflecting the creation of a free particle—here, the preparation of particles considered later to scatter—would involve the clear violation of applicable fundamental conservation laws, cf. e.g., [27], pp. 29–30. The salient point is that under the orthodox interpretation of quantum theory (which incorporates the eigenvalue–eigenstate link), if the classical correspondent of an eigenvalue is directly observed in an appropriate experimental apparatus then credence is lent to the proposition that the system was prepared in a state in which the associated field property (excitation) obtains, but the mathematical application itself of the operator $a_{k,\alpha }^{†}$ to a state-vector does not correspond to any such preparation.

It is generally assumed by those of the opinion that particles exist that fields do as well; indeed, for example, it is even held that “the fields that enter into our theories are put there mainly for the purpose of making quanta—making particles, that is; and for intermediating particle interactions; or in some cases for acquiring non-zero vacuum expectation values to break symmetries; or to enter into local gauge transformations; and so on” [28]. The proper context of the question of the existence of virtual particles over and above other particles is that of *scattering*, where they play the role of force mediators, and *decay processes* rather than in the mathematical representations of non-interacting fields. The presence of these particles is reflected in empirical data such as scattering cross-sections. Accordingly, Teller argues differently from Weingard (in relation to i) against the inference to the existence of virtual particles from the role of raising (creation) and lowering (annihilation) operators by instead and more pertinently focusing on naive interpretations of the Feynman diagrams, commonly involved in the calculation of such quantities, which he takes “to be misleading in the extreme” ([29], p. 137).

If understood as precise space-time diagrams, Feynman diagrams suggest to some the creation of virtual particles at specific space-time points because of their inclusion of vertices: in these diagrams, a vertex is a (graphical) point. However, as Teller correctly notes, “there is no strong reason” for interpreting the association of these operators with vertices as representing creation and annihilation, particularly ones occurring at specific space-time points *x* (cf. [29], p. 139). However, he then argues further that this is so because quanta cannot be endowed with “primitive thisness” (*haeccity*) as they are represented naturally via the Fock formalism, which incorporates their statistical properties in collectives, rendering that implausible. However, then the argument is *de facto* made against inferring the creation of *particles in general* as indicated by these diagrams rather than against inferring the creation/annihilation of virtual particles *per se*, because particles associated with such vertices may also be the particles later detected, so does not tell against the position (IV) being defended currently. (Note nonetheless that the requirement of “primitive thisness” is widely considered irrelevant to the modern particle concept, cf. [6,7]). It is the rejection of an overly simplistic physical interpretation of events only abstractly portrayed in Feynman diagrams that is relevant here which, although correct, is beside the point of the existence of virtual particles themselves as distinguished from generic particles. The discussion mainly indicates the folly of using individual Feynman diagrams as direct, specific, literal physical portrayals of spatio-temporal processes.

In the same context, Teller also argues that any talk of ‘state transitions’ or ‘quantum (state) jumps’ to intermediate states is misleading, as it is only upon measurement that a quantum pure state is reached in any discontinuous manner—in particular, not in the middle of the time evolution (in the Schrödinger picture) starting from some initial pure state—because “With interaction this pure state evolves, smoothly and deterministically, into a state superposition of eigenstates of other eigenstates of the observable in question”, [29], p. 138. It is his view that “raising and lowering operators at least describe what happens in measurement…in the uninteresting sense that they can be used to describe the eigenstates that result when a measurement takes place” but even then do not describe “in any more enlightening way…what goes on when we make a measurement”, nor is there any “reason to think that the raising operators describe a state-transition process” upon pair creation. These points are valid with respect to the quantum state, as noted above, but would only have force in the argument against virtual particles were the raising and lowering operators appearing in the quantum probability calculation to be strongly ontologically interpretable; the presence of raising and lowering operators in calculations of transition probabilities need not be directly associated with discontinuous state change, i.e., state jumps, but only mathematically indicate the presence of possibilities of them by virtue of their contribution to the overall quantum state-transition probabilities. Once again, the argument does not tell against Position IV.

Let us move on to the related “superposition argument”, which is generally regarded as the most powerful of arguments against the existence of virtual particles. Weingard argued that such particles do not exist because neither the number nor kinds of virtual particles are *sharp* in “the superposition represented by…field amplitude 〈f|S|i〉”, where *i* and *f* indicate the initial and final states and the scattering operator is

$$S=\sum_{k}S_{k}\;,$$

the last being proportional to a product of interaction Lagrangians (space-time integration suppressed here) [30,31]. “It is not only that our amplitude 〈f|S|i〉 is a superposition," but that many terms are present in addition to one involving a specific number of particles and that S|i〉 “contains not only terms contributing to 〈f|S|i〉 but also the terms relevant to all other possible final states”: Until a final measurement is made, the final state *f* is *indefinite*—“…no definite virtual process has taken place, the measurement brings it about that” the final state is the specific one indicated above [31].

Weingard then correctly points out that this situation is analogous to that in ordinary quantum mechanics when the system state is a superposition of eigenstates of an observable of interest. However, these characteristics of the quantum state are uncontroversial elements of the understanding of quantum physical processes in general (apart, perhaps, from in the macroscopic realm, which is not the subject of investigation here) and, like such superpositions in general, this particular superposition does not pose a problem for the existence of the system involved, the quantum state, or field aspects corresponding to particles: Whenever any well performed measurement of the system is made, a value of what is measured is found that is attributable to that system, and there is always a well-defined expectation value for the number of quanta to be found; the system itself does not cease to exist meanwhile nor does its state, however unsharp it might be with regard to the quantity in question, here the system being the collection of quantum fields and their particles/quanta; the effect of such (sharp) measurement is to only sharpen this value; cf. [32,33].

This situation was said to be directly analogous, in particular, to the situation in the famous double-slit experiment in ordinary, non-relativistic quantum mechanics wherein a quantum system is prepared on one side of a double-slitted diaphragm and detected on the other side (about which, cf. e.g., [34]). This analogy does not strengthen the argument against virtual particles but serves only to emphasize that: (a) the presence of intermediate state amplitudes—the states analogous to the states involving virtual particles—does have observable implications, namely, quantum interference (significant, for example, in the case of pair creation), and (b) these cannot be disregarded, because the final observed states cannot be reached without with there being a non-zero probability of the system being found in each of them, and it is *certain* that it is to be found (however counterfactually) in one of the states of the collective they form, in the sense that *if* the position at the diaphragm *were* to be measured the system, it will with certainty be found in *a* slit. Similarly, when a measurement for a state containing a particle of a given type is made, an ostensibly virtual particle may be found and, then, uncontroversially considered real and present to anyone accepting the existence of particles at all. Such a scenario appears within the context of the photon delayed-choice experiment (discussed in Section 2) as the second of the two alternative eventualities (to the one mentioned there): if measured *before* (re)absorption an ostensibly virtual particle is considered real.

Now let us consider some other objections to the thesis that virtual particles are as real as others that arise from other perspectives regarding virtuality, some of which are discussed in the Introduction and Section 2. Recall that the standard means of distinguishing between these two sorts of particle is via the form of line used to represent one in Feynman diagrams which are computational tools productively used to represent the various and infinite number of terms involved in the perturbation-theoretical calculations of outcomes of scattering experiments that are the mainstay of the investigation of particle physics: The particles represented by internal lines are considered virtual while the others are represented by external lines, with the more precise meaning that in the former, a specific sort of propagator appears (e.g., according to the corresponding Feynman rules) in the terms of perturbation-theoretical expansions of the contributions to the scattering probability for a given transition between asymptotic initial and final states. Accordingly, any particle not eventually appearing as a quantum of a state of any free field is virtual. However, as noted, for many, if not all, sorts of particle that can appear as a free particle, there are circumstances in which that particle can appear as a virtual particle (i.e., a quantum associated with a distinct, mediating field). Therefore, the distinction is not a fundamental one and any objection on this basis to Position IV fails.

Another objection arises as follows. For reasons related to the mathematical form of propagators, as mentioned in Section 2, it is sometimes incorrectly argued that if virtual particles exist then the energy-momentum relation

(4) $$E^{2}=p^{2}+m^{2}$$

(where *c* is taken to be unity and *p* is momentum, that is, $k^{2}\neq m^{2}$, where *m* is the standardly attributed mass value and *k* is the four-momentum) must fail at a vertex of a Feynman diagram that includes an internal line. This relation would appear to be violated in scattering processes in some perturbation-theoretical diagrams, but Feynman’s approach to such diagrams allows the mass to differ from the standard value to satisfy this relation at vertices, enforcing this relation. (It is noteworthy also that such a vertex refers to no one specific space-time point but rather is one that ranges over all space and time; the diagram in which it appears is not a proper space-time diagram and the vertex location is integrated over all of space and time in performing the calculations in which it appears).

Any apparent violation can also be understood as due to virtual particles’ possessing some of the potential energy that classically would be associated with the fundamental force involved in any such interaction, as Anthony Sudbery has pointed out. “The only nonconservation in these processes is of *kinetic energy*; as in classical mechanics, the total energy is the sum of the kinetic and potential energies and the principle of conservation of energy holds exactly at all times” [35], p. 27. In addition, even if the idea of some conservation law violation were to be accepted, it is still the case that all other rules associated with conservation laws but this one are strictly obeyed everywhere, thereby preserving other aspects providing the quantum’s identity, and, most importantly that all conservations laws are certainly obeyed *in the overall scattering process*, which is *all that quantum theory ever makes precise empirical predictions about*. This reflects fundamental differences between quantum theory and classical theory: In quantum theory, predictions of observed quantities are probabilistic-statistical, and pictorial representations of objects and phenomena of the kind familiar from the large-scale world of common-sense are almost never literally interpretable, including corresponding naive pictures of particles and fields.

In Feynman’s way of diagramming, which enables an automatically covariance-preserving way of organizing the terms of the associated perturbation-theoretical calculations, there is not even the appearance of the violation of energy conservation: There, the mass–energy *m* of the associated virtual particle(s) always takes precisely the value needed for the energy conservation relation to be obeyed at each vertex, and all other conservation law expressions also always hold exactly. The virtual particle mass becomes a variable of integration (cf., [34]) and the particle is said to be “off mass-shell,” which as mentioned previously is for some an alternative indication of virtuality; any difference of mass of virtual particles from standard values in intermediate states is not physically problematic but only requires caution against incorrectly attributing the free-state values of masses given to particles while they are in those short-lived states.

A yet additional argument offered against assenting to the existence of virtual particles can also be briefly mentioned here, one relating to ontological commitment and explanation, namely that virtual particles should not be taken to exist because, it is claimed, *they are not needed* for the explanation of any physical phenomenon; taking them to exist is argued to be a violation of ontological parsimony. Although the question of the applicability of the principle of parsimony to physical theory is beyond the scope of the current analysis, the explanatory value of the notion of virtual particles is arguably equal to that of other particles in RQFT, something which provides support for the reality of the virtual elementary particles and Position IV rather than contradicts it.

## 6. Virtual Particles are as Real as Others

The explanatory value of virtual particles and processes involving them was already to some extent attested in Section 2. In addition to the points mentioned there, it is the case that the explanation of the observed values of mass and charge differing from their “bare” theoretical values is also considered explicable via RQFT in a way crucially involving virtual particles. As Yuval Ne’eman has put it, upon its introduction, the theory of QED “provided the means of including in the calculations the additional effects due to virtual processes, i.e., processes that can take place beneath the threshold of observability set by the principle of uncertainty; for example, the emission and subsequent reabsorption of photons, which is going on all the time, making up what is usually called a ‘photon cloud’ surrounding the electron in the quantum world. How impressive, when the new theory gave the value of the electron’s magnetic moment “*g*-2” to the tenth digit, and this with a perfect fit with experiment!…The theory aimed at presenting the proper quantum treatment for electromagnetic fields and electric charges, and was based on an idea proposed by Dirac in 1927: to treat the electromagnetic field itself not as a continuous quantity but as something composed of discrete entities or quanta …Even today it is considered to be the most precise existing scientific theory” [36], p. 59.

Another important example of the explanatory value of virtual particles is that of the exploration and elucidation of the internal structure of protons: The probing of proton structure is done primarily not by initially free electrons or other particles, but rather by using *virtual photons*: “‘Photographing’ an object by scattering an electron beam off it is a well-proved technique in physics” ([19], p. 172). “Having measured the size of the proton [with an electron beam],…[we] take a look at its structure by increasing the [momentum] of the [virtual] photon to give better spatial resolution” ([19], p. 179). “[T]he important issue is what happens…[in deep inelastic scattering when a high-energy] (virtual) photon interacts with a proton. The role of the electron beam is simply that it is responsible for the presence of the virtual photon” ([19], p. 184). This method of analysis produces experimentally confirmed predictions for cross-sections for virtual photon–proton and similar scattering processes ([19], Chapter 10). Here, there is no question of the presence and, indeed, the relative primacy of the virtual particle for the purpose of making experimental observations: The goal is to perform a scattering experiment with high-energy photons, and virtual ones that happen incidentally to have been created using an electron beam serve this purpose most effectively. Indeed, such use of elementary particles turns on its head the idea of the secondary status of virtual particles in comparison to other particles sometimes supposed to be “more real”.

Each sort of virtual particle can be considered an “excitation of a quantum field” (more accurately, a quantum of energy associated with the field) with defining characteristics that are the same whether it or not it is in the virtual ’version’ and allow it to be used in theoretical explanations of physical phenomena. Indeed, this is why, for example, a virtual particle X is considered an X and *not some other particle* Y; for example, the electron and the virtual electron are identical in defining characteristics whereas the electron and other leptons—such as the muon which, moreover, has its own virtual ‘version’—are not. Fundamentally, particle identity in no way depends on the choice of diagrammatic representation of interactions in performing calculations of transition probabilities—whereas whether a quantum appears as an internal line in a Feynman diagram does—or even on the physical circumstances (such as how far it has propagated or encountering another particle) in which it appears. What is the virtual particle outside the context of perturbation theory where particles are indicated by lines in diagrams? Such a particle is a quantum, associated with a specific force-mediating field, indicated in a non-trivial state amplitude within the interval of time during which scattering or particle decay takes place, that is, between the directly measured states. This novel, more general characterization of virtual particle applies to the examples of virtual particles provided here above while avoiding the perils and potential inconsistencies in application of previous characterizations, including the heretofore standard one, which depend on the context of perturbation theory to indicate virtuality, and it provides a realist meaning to the notion of virtual particle. Under this more objective characterization, virtual particles are as real as all others.

One may note also that one method of non-perturbative field theory, lattice quantum chromodynamics, treats virtual particles differently from others, as “links” between sites of a discrete space-time lattice at which interacting particles are located, (in the limit taken) infinitesimally closely without a finite interval in which to propagate. However, no matter what calculational technique might be invoked for predicting measurable quantities in the context of standard QFT, the physics involves processes taking place within the interval between the preparation of the initial state and the measurement of the final state. The intermediate-time states will non-trivially include amplitudes involving the presence of mediating quanta (were they to be directly measured within what would be their relatively short lifetime); these will correspond to quanta that may be labeled as virtual (according to the standard definition) only because they do not “appear” in the final, measured state. More significantly from the point of view of QFT, the relatively brief presence and propagation of virtual quanta of mediating fields distinct from those of interacting particles (e.g., a proton and electron in an electromagnetic scattering process) precludes action-at-a-distance between those directly observed particles, in that regard rendering the virtual particles indispensable.

## 7. Conclusions

The question of whether virtual particles exist, like other particles in a world described by quantum field theory, is considered above in light of discussions of fundamental interactions and their previous critical analysis. Its resolution depends on the clarification of the nature and role of mathematical calculations and their elements used in the description, prediction, and explanation of associated physical effects. It is shown that the arguments against the existence of virtual particles, which depend on a putatively fundamental distinction from other elementary particles, fail to establish that they are less real than other particles because their distinction is dependent on circumstance and the methodology of analysis selected—most prominently, the use of perturbative methods and associated diagrammatics in the calculation of transition probabilities and the potentially misleading graphical character of the latter.

Here, a characterization of virtuality in terms of intermediate states is provided that is independent of perturbation theory and includes the standardly recognized virtual particles. According to it, there is no inherent, objective and fundamental difference *qua* particles (field quanta) between the virtual and other particles; in the language of Feynman diagrams in which this distinction is standardly made, in some circumstances an instance of any given species of particle corresponds to an internal line and in other circumstances an external line. This is most vividly seen by consideration in differing possible circumstances of the photon, which is capable of mediating interactions across extremely large scales and, in the limiting case, freely propagating indefinitely; the (non)presence of a charged particle and the concomitant theorist’s *choice* of method used to explain phenomena, for example, using perturbation theory can together decide whether a particle is considered a virtual one. Therefore, the distinction of virtual particles from others is ontologically irrelevant.
